# Rice husk hydrochars from metal chloride-assisted hydrothermal carbonization as biosorbents of organics from aqueous solution

**DOI:** 10.1186/s40643-021-00451-w

**Published:** 2021-10-09

**Authors:** Yin Li, Fana Mulugeta Hagos, Rongrong Chen, Hanxin Qian, Chengxing Mo, Jing Di, Xikun Gai, Ruiqin Yang, Genxing Pan, Shengdao Shan

**Affiliations:** 1 Zhejiang Provincial Key Lab for Chemical and Biological Processing Technology of Farm Product, School of Biological and Chemical Engineering, Zhejiang University of Science and Technology, Hangzhou, 310023 Zhejiang China; 2 Institute of Resource, Ecosystem and Environment of Agriculture, Nanjing Agricultural University, 1 Weigang, Nanjing, 210095 China; 3 Key Laboratory of Recycling and Eco-Treatment of Waste Biomass of Zhejiang Province, Zhejiang University of Science and Technology, Hangzhou, 310023 Zhejiang China

**Keywords:** Char, Biomass, Hydrothermal carbonization, Adsorption, Organics

## Abstract

Hydrochar a carbon-rich material resulting from hydrothermal carbonization of biomass, has received substantial attention because of its potential application in various areas such as carbon sequestration, bioenergy production and environmental amelioration. A series of hydrochars were prepared by metal chloride-assisted hydrothermal carbonization of rice husk and characterized by elemental analysis, zeta potential, X-ray diffraction, Brunauer–Emmett–Teller measurements, Fourier transform infrared spectroscopy, thermogravimetric analysis, X-ray photoelectron spectroscopy and scanning electron microscopy. The results reveal that the prepared hydrochars have carbon contents ranging from 45.01 to 58.71%, BET specific areas between 13.23 and 45.97 m^2^/g, and rich O-containing functional groups on the surfaces. The metal chlorides added in the feedwater could improve the degree of carbonization and show significant effects on the physical, chemical and adsorption properties of the hydrochars. The adsorption of the selected organics on the hydrochars is a spontaneous and physisorption-dominated process. The hydrochars possess larger adsorption capacities for 2-naphthol than for berberine hydrochloride and Congo red, and the modeling maximum adsorption capacities of 2-naphthol are in the range of 170.1–2680 mg/g. The adsorption equilibrium could be accomplished in 10, 40 and 30 min for 2-naphthol, berberine hydrochloride and Congo red, respectively. These results suggest metal chloride-assisted hydrothermal carbonization a promising method for converting biomass waste into effective adsorbents for wastewater treatment.

**Supplementary Information:**

The online version contains supplementary material available at 10.1186/s40643-021-00451-w.

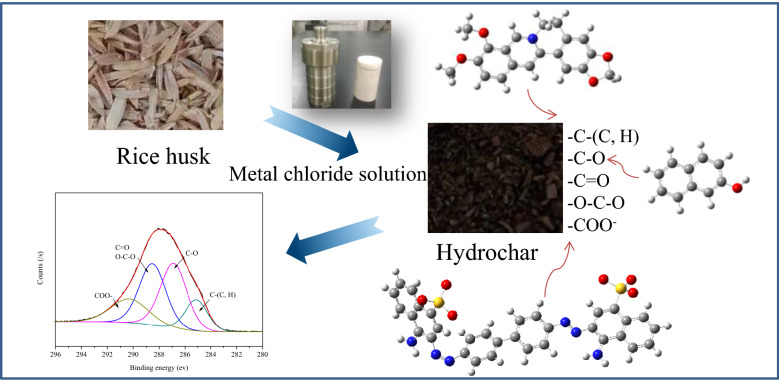

## Introduction

Agricultural waste biomass is an abundant, renewable but still largely underutilized resource worldwide (Bian et al. 2019). Direct burning or discarding agricultural residues on the field could cause severe environmental issues, thus the management of agricultural waste is a major environmental concern, especially in the countries with large agricultural production, and recycling is one of the economically viable and sustainable options (Campos et al. 2020). On the other hand, increasing anthropogenic activities and expansion of synthetic industrial chemical manufacturing have led to the release of organic pollutants into surface and ground water resources (Wang et al. 2019). These pollutants such as dyes, antibiotics and aromatic compounds discharged from textile, agrochemical, and pharmaceutical industry are known to be highly toxic, chemically stable, not readily biodegradable in water, can enrich in food chain, and present potential threats to environment and human health (Drout et al. 2019; Grandclément et al. 2017). Therefore, effective treatment of waste water containing organic pollutants, especially persistent organics, has received wide attention.

Adsorption has been widely applied in contaminant removal from water due to its cost effectiveness, high efficiency and easy operation (Jian et al. 2018; Tong et al. 2019). Hydrochar is a solid carbon-rich product generated from hydrothermal carbonization (HTC) of biomass at a moderate carbonization temperature (180–350 °C) and autogenous pressure in the presence of water (Li et al. 2019a), and has been applied as a sorbent for contaminant immobilization and removal in soil and water (Khan et al. 2019). Compared to biochar from pyrolysis of biomass, hydrochar could present considerably higher adsorption capability for organic pollutants due to the oxygen-rich functional groups on its surface (Hairuddin et al. 2019; Kambo et al. 2015; Zhang et al. 2021). Furthermore, hydrothermal carbonization can handle feedstocks with high moisture contents, and the liquid and gaseous products obtained from HTC procedure such as bio-oil and syngas could be utilized as fuels or the sources of platform molecules (Wang et al. 2020). These make HTC a promising method of converting agricultural waste biomass into low-cost hydrochar adsorbents.

During the HTC process of biomass, additives in feed water could alter the pathway of the reactions involved and accelerate the reaction rate (Khan et al. 2019), thus influence the physico-chemical and adsorption properties of the obtained hydrochars. Chemical reactions involved in a biomass HTC process include hydrolysis, dehydration, decarboxylation, polymerization, condensation, and aromatization, and the addition of acids, alkalis and salts in HTC has been suggested to catalyze the hydrolysis, dehydration and carbonization processes and tailor the reaction path for achieving desirable products (Chen et al. 2017; Flora et al. 2013; Khan et al. 2019; Reza et al. 2015; Wang et al. 2010). Metal ions have been reported to be able to significantly facilitate the hydrolysis of biomass in the presence of ionic liquids (Wang et al. 2014; Wiredu et al. 2015), and metal chlorides can accelerate the degradation of hemicellulose and cellulose and the decomposition of glucose in hot compressed water simultaneously (Lopez-Linares et al. 2013; Ma et al. 2010). On the other hand, metal chlorides such as FeCl_3_ and ZnCl_2_ are widely used as chemical activating agents in additional activation steps to increase the porosity and surface area of biochar and hydrochar for the production of activated carbon (Pezoti et al. 2014; Zyoud et al. 2015), and the direct addition of metal chlorides in biomass pyrolysis process could also improve the specific surface area of the carbon residue (Lugovoy et al. 2019). However, only few literatures reported the influence of inorganic salts on hydrothermal carbonization of biomass (Lynam et al. 2011, 2012), and the effects of metal chlorides on physico-chemical and adsorption properties of hydrochars need to be further explored.

In this study, rice husk (RH), a lignocellulosic agricultural waste, abundantly available, was used as raw material to prepare hydrochars as potential adsorbents through a metal chloride-assisted hydrothermal carbonization process. The influence of metal chlorides, KCl, CaCl_2_ and FeCl_3_ at different concentrations in the hydrothermal medium on the physico-chemical properties of rice husk hydrochars was explored. 2-Naphthol, berberine hydrochloride (BH) and Congo red (CR) were selected as model compounds of aromatics, antibiotics and dyes, respectively, and the adsorption abilities of the hydrochars for these organic pollutant model compounds from aqueous solutions were investigated.

## Materials and methods

### Chemicals

Rice husk (with average moisture content of 16 wt%) was collected from Zhejiang province, China. KCl, CaCl_2_, FeCl_3_ and 2-naphthol were provided by Shanghai Lingfeng Chemical Reagent Co., Ltd.; BH was purchased from Shanghai Darui Fine Chemicals Co., Ltd.; and CR was obtained from Tianjin Zhiyuan Chemical Reagent Co., Ltd. All the chemicals are AR grade. The formula, chemical structure and molecular weight of 2-naphthol, BH and CR are listed in Additional file 1: Table S1.

### Hydrochar preparation

Hydrothermal carbonization was performed in a 100-mL autoclave reactor with 5 g of rice husk and 40 mL of metal chloride solution added in the Teflon insert. KCl, CaCl_2_ and FeCl_3_ with concentrations of 0.2, 0.6 and 1.0 mol/L were selected to evaluate the effects of metal chloride additives. The reactor was tightly closed, heated to 200 °C and maintained for 7 h, then cooled at room temperature. Hydrochar, the solid product, was collected by filtering the obtained mixture. The original hydrochar was treated with the procedure described in our previous work to remove impurities from its surface and obtain dry hydrochar sample (Li et al. 2018). The hydrochar yield (%) was determined as the weight of dried hydrochar to the weight of dry rice husk.

The hydrochar samples were designated as RHH, RHHK0.2, RHHK0.6, RHHK1, RHHCa0.2, RHHCa0.6, RHHCa1, RHHFe0.2, RHHFe0.6, RHHFe1, where RHH is the abbreviation of rice husk hydrochar, K, Ca and Fe indicate the metal chloride added to the medium and the suffix number means the concentration.

### Hydrochar characterization

Ash content was determined by heating the hydrochar sample in a muffle furnace at 550 °C for 3 h, and the value was the ratio of ash weight to dry hydrochar weight. Elemental (C, H, N, S) analysis was carried out on a Vario Micro Cube elemental analyzer, and O content was the difference between the amount of C, H, N, S, ash content and the hydrochar dry mass. Zeta potential of the samples in pure water was determined on a Zetasizer Nano Series zeta potential analyzer. X-ray diffraction (XRD) patterns were performed using a Rigaku Ultima IV diffractometer with a CuKα radiation (40 kV, 20 mA). Fourier transform infrared (FTIR) spectrometer (Bruker Vertex 70) was used to identify the surface functional groups on the hydrochar surface. Brunauer–Emmett–Teller (BET) surface area, pore volume and average pore diameter of the samples were measured by N_2_ adsorption–desorption at 77 K on a Sorptometer Quantachrome Autosorb iQ apparatus. Thermogravimetric analysis (TGA) was carried out on a NETZSCH STA 449F3 thermal gravimetric analyzer to evaluate the composition and stability of the hydrochars. The surface chemical compositions of the materials were characterized through X-ray photoelectron spectroscopy (XPS) via a Thermo Scientifc K-Alpha spectrometer. A Hitachi S3700 scanning electron microscope (SEM) was used to observe the surface morphology of the hydrochars.

### Adsorption thermodynamics

For each set of adsorption isotherm experiment, 30 mL of organic solution with known initial concentration, *C*_0_(mg/mL), was mixed with 0.06 g of the hydrochar in a 100-mL stoppered triangle flask and agitated in a shaker at 170 rpm at 298, 308 or 318 K for 8 h. The equilibrium concentration, *C*_e_ (mg/mL), of CR, BH and 2-naphthol in the solution was determined on a UV–vis spectrophotometer at 499 nm, 345 nm and 274 nm, respectively. Equilibrium adsorption capacity *Q*_e_ (mg/g) was calculated by:

1 $$Q_{e} = \left( {C_{0} - C_{e} } \right) \cdot V/m,$$

where *V* (mL) is the solution volume and m (g) is the dry weight of the hydrochar.

Langmuir and Freundlich models given by the following equations were used to fit the experimental data:

Langmuir equation:

2 $$Q_{{\text{e}}} = \frac{{Q_{{\text{m}}} K_{{\text{L}}} C_{{\text{e}}} }}{{{1 + }K_{{\text{L}}} C_{{\text{e}}} }}.$$

Freundlich equation:

3 $$Q_{{\text{e}}} = K_{{\text{F}}} C_{{\text{e}}}^{{{1/}n}} ,$$

where *Q*_*m*_ (mg/g) is the maximum monolayer adsorption capacity, *K*_*L*_ (mL/mg) is the Langmuir constant related to the adsorption energy. *K*_*F*_ ((mg/g)(mL/mg)^1/n^) and 1/n are the Freundlich constants indicating the adsorption capacity and intensity, respectively.

### Adsorption kinetics

Triangle flasks containing 0.06 g of selected hydrochar and 30 mL of 0.5 mg/mL adsorbate solution in each were shaken at 170 rpm and 298 K for 10, 20, 30, 40, 50, 60, 90, 120, 180 and 240 min. The concentration of the organics in the liquid phase at time t, *C*_t_ (mg/mL) was analyzed, and *Q*_t_ (mg/g), adsorption capacity at t was calculated using:

4 $$Q_{t} = \left( {C_{0} - C_{t} } \right) \cdot V/m.$$

Pseudo-first-order and pseudo-second-order adsorption kinetic models were used in the forms as follows to fit the experimental kinetic data:

Pseudo-first-order equation:

5 $$Q_{{\text{t}}} = q_{{\text{e}}} (1 - e^{{ - k_{1} t}} ).$$

Pseudo-second-order equation:

6 $$Q_{{\text{t}}} = \frac{{tk_{{2}} q_{{\text{e}}}^{{2}} }}{{tk_{{2}} q_{{\text{e}}} + 1}},$$

where *q*_e_(mg/g) is the calculated equilibrium adsorption capacity, *k*_1_ (1/min) and *k*_2_ (g/(mg·min)) are the adsorption rate constants of the pseudo-first-order equation and pseudo-second-order equation, respectively, and the initial adsorption rate is calculated through $v_{{0}} = k_{{2}} q_{{\text{e}}}^{{2}}$.

## Results and discussion

### Characterization of hydrochars

Table 1 shows the main physico-chemical characteristics including yield, ash, zeta potential, elemental content, and pore properties of the rice husk hydrochars prepared in this study. The hydrochars prepared with metal chlorides added in the feed water display lower yields (28.97% to 47.02%) as compared to the hydrochar prepared in pure water (RHH, 47.10%), and the values decrease contrariwise to the concentration of the metal chlorides. The HTC process of lignocellulosic biomass contains a complex reaction network, hemicellulose, cellulose and lignin undergo a series of reactions starting from hydrolysis, followed by dehydration and decarboxylation to yield water-soluble intermediates like furfural, 5-hydroxymethylfurfural (HMF) and phenolic derivatives, and hydrochar is formed from both the polymerization of the reactive intermediates and solid–solid conversion of the solid residues (Khan et al. 2019). The above results indicate that the addition of metal chlorides on the HTC process should be able to facilitate the reactions that can cause mass loss of the final hydrochar, such as organic matter decomposition to produce water-soluble low molecular products and solid–solid conversion of the solid residues, and the greater the metal chloride concentration, the higher the catalytic activity. Furthermore, different metal ions present obvious different influences on the hydrochar yields. At the same concentrations of added metal chlorides, RHHFes exhibit the lowest yields among all the hydrochar samples, while RHHCas display the highest yields. Some inorganic salts have been reported to be able to significantly catalyze the degradation of hemicellulose and cellulose in the HTC process, while FeCl_3_ shows a particularly strong activity (Liu et al. 2009), and pretreating biomass with FeCl_3_ can alter the structure of lignocellulose matrix and improve sugar yields in subsequent enzymatic hydrolysis (Lopez-Linares et al. 2013). Additionally, among the three chloride solutions, FeCl_3_ solution presents the highest acidity, which could further accelerate the hydrolysis of cellulose and hemicellulose (Rabemanolontsoa and Saka 2016; Reza et al. 2015). These might be the reasons for the low yields of RHHFes. On the other side, it was reported that Ca^2+^ cation could promote glucose conversion into HMF (Torres-Olea et al. 2021), which is a highly reactive intermediate in condensation reactions to form hydrochar, and this might result in the higher yields of RHHCas. Furthermore, the yield of the hydrochar prepared with monovalent salt KCl, divalent salt CaCl_2_ and trivalent salt FeCl_3_ added in the feed solvent at the same concentration does not present a linear correlation with the ionic strength of the feed solution, indicating that the yield of the metal chloride-assisted hydrochar is mainly affected by the metal salt type rather than the ionic strength of the medium, and the combined effect of the metal cation’s catalysis and solution acidity on biomass decomposition and hydrochar formation reactions may determine the final yield of the hydrochar.

**Table 1:** Yields, ash contents, compositions and pore properties of the rice husk hydrochars

Sample	Yield (%)	Ash content (%)	Zeta potential	Elemental composition								Pore properties		
				C (%)	H (%)	O (%)	N (%)	S (%)	H/C	O/C	(O + N)/C	BET surface area (m^2^/g)	Pore volume (cm^3^/g)	Pore diameter (nm)
RHH	47.10	10.43	− 19.5	45.01	5.04	38.35	1.13	0.04	1.34	0.64	0.66	13.83	0.04	3.07
RHHK0.2	44.85	7.00	− 21.1	48.44	5.19	38.50	0.82	0.05	1.29	0.60	0.61	13.23	0.39	3.24
RHHK0.6	43.48	7.90	− 27.2	53.33	5.48	32.22	1.00	0.07	1.23	0.45	0.47	16.33	0.06	3.72
RHHK1	38.83	8.20	− 32.8	52.93	5.09	32.61	1.02	0.15	1.15	0.46	0.48	18.04	0.05	2.96
RHHCa0.2	47.02	10.80	− 10.9	51.26	5.18	31.77	0.93	0.06	1.21	0.46	0.48	13.75	0.05	3.52
RHHCa0.6	43.09	14.67	− 16.5	58.30	4.64	21.14	1.17	0.08	0.96	0.27	0.29	37.58	0.14	4.18
RHHCa1	42.10	15.00	− 20.1	58.25	4.56	20.81	1.28	0.10	0.94	0.27	0.29	45.97	0.23	5.43
RHHFe0.2	30.59	13.28	− 12.2	58.71	4.65	22.55	0.73	0.08	0.95	0.29	0.30	45.16	0.24	5.87
RHHFe0.6	29.17	15.00	− 25.7	53.09	4.06	27.30	0.46	0.09	0.92	0.39	0.39	42.26	0.23	5.94
RHHFe1	28.97	15.40	− 36.2	54.47	3.96	25.65	0.40	0.12	0.87	0.35	0.36	36.27	0.22	6.55

From Table 1, it is not surprising to see the higher ash contents of RHHCas (10.80% to 15.00%) and RHHFes (13.28% to 15.40%) than RHH (10.43%) since their greater organic matter loss from biomass decomposition in the HTC process, and this result agrees with the yield of these samples. Additionally, ash could also be introduced by the deposition of salts on the hydrochars (Fedotov et al. 2014). KCl is more soluble when compared to CaCl_2_ and FeCl_3_, and thus most of the K^+^ would probably distribute in the aqueous phase while the other two metal cations can bind more strongly to the char matrix and lead to the higher ash contents of RHHCas and RHHFes. On the other hand, it is interesting to notice that RHHKs have lower ash contents (7.00% to 8.20%) than RHH. Since there is no evidence that KCl could improve the dissolution of silica, the main component of rice husk ash (Omatola and Onojah 2009), into hot compressed water, further investigations will be needed to explain this result.

The main components of the rice husk hydrochars are carbon (45.01–58.71%) and oxygen (35.81–45.50%) according to the elemental analysis results listed in Table 1. The higher C contents and lower O contents of RHHKs, RHHCas and RHHFes than RHH indicate that adding metal chlorides in feed water could improve the carbonization degree of the final hydrochar. Chloride ions can disrupt cellulose hydrogen bonding to facilitate its solubilization and removal from biomass (Lynam et al. 2012), and the remaining lignin should have a higher degree of aromatization. Moreover, chloride anions might also serve as a catalyst to crosslink hemicelluloses and cellulose and thus eliminate O component from the hydrochar products (Lynam et al. 2011). On the other hand, metal salts could catalyze condensation reactions which could cause the formation of highly aromatic products (Lynam et al. 2012), and metal salt pretreatment before pyrolysis was proven to be able to increase the aromatization of biochar products (Xiao et al. 2018). These might lead to the higher carbonization degree of the rice husk hydrochars from metal chloride-assisted HTC. Furthermore, Lewis acid metal salts such as CaCl_2_ and FeCl_3_ in water could further benefit the extent of hydrolysis and dehydration reactions to produce water-soluble acids and HMF (Degirmenci and Hensen 2014; Wang et al. 2015), meanwhile the polymerization of HMF results in aromatic carbon networks, and these might result in the even lower O contents of RHHCas and RHHFes. (O + N)/C is an indicator of polarity, and a greater value indicates a greater polarity (Tan et al. 2020). It could be observed that all the hydrochars produced from metal chloride-assisted HTC display lower polarity than RHH, indicating a higher hydrophobicity of these hydrochars’ surfaces. On the other hand, RHHCa0.6, RHHCa1 and RHHFes clearly exhibit the lowest polarity among all the hydrochar samples due to their high carbonization degree.

The zeta potential results (Table 1) show that the surface charge of all the hydrochars is moderately negative, consistent with results of reported hydrochars (Han et al. 2020; Tan et al. 2020). The values are in the range of − 10.9 to − 36.2 mV, and the negative surface charge increases with increasing concentration of the metal chlorides. These might be attributed to the combined effects of ash content, O-containing surface functional groups, polarity and H-bonds on the surface charge of the hydrochars, in which the first three should have positive effect on negative charges, whereas H-bonds could provide negative effect on it (Tan et al. 2020). The surface negative charge should benefit the electrostatic attraction of the hydrochars for organic cations.

**Fig. 1:**
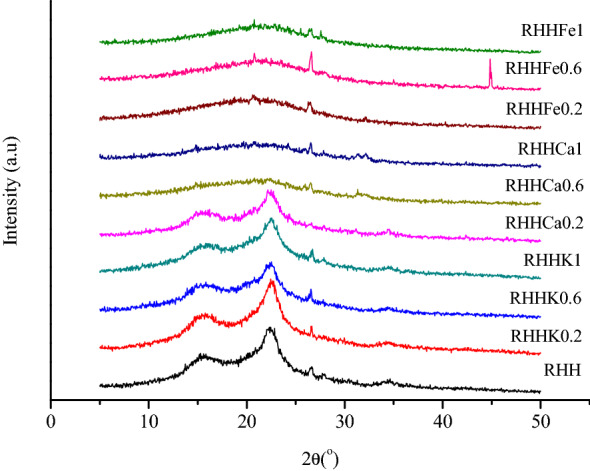
XRD pattern of the rice husk hydrochars

The XRD pattern of the rice husk hydrochars in the range of 5–50° (2θ) is depicted in Fig. 1, suggesting the coexistence of crystalline and amorphous phases. The peaks in the diffractograms of RHH, RHHKs and RHHCa0.2 at 2θ = 16° and 22° corresponding to cellulose structure (Liu et al. 2020) could be attributed to the incomplete carbonization of these samples. While these two peaks disappeared in the XRD profiles of RHHCa0.6, RHHCa1 and RHHFes, and a broad peak could be observed in these hydrochars located between 21° and 23° (2θ), suggesting the complete decomposition of rice husk cellulose to produce amorphous carbon. These results further confirm the stronger catalytic activities of FeCl_3_ and CaCl_2_ at higher concentrations, and are in accordance with the yields and the elemental compositions of the hydrochars. On the other hand, the small peak at 2θ = 26.5° could be associated with the graphite structure (Navarro-Suarez et al. 2014) of the hydrochars.

As can be seen from Table 1, the BET surface areas, pore volumes and average pore diameters of the hydrochars were determined to be in the range of 13.23–45.97 m^2^/g, 0.04–0.39 cm^3^/g, and 2.96–6.55 nm, respectively. Generally, the hydrochars prepared with metal chlorides added in the feed water present higher BET surface areas and larger pore volumes than RHH prepared in pure water, in accordance with the lower yields of these hydrochar samples and further confirming the positive effect of the metal chlorides on the decomposition of rice husk biomass in the HTC process. Furthermore, RHHCa0.6, RHHCa1 and RHHFes provide much higher BET specific surface areas, greater pore volumes and also larger pore sizes than the other hydrochar samples, and the pore size of RHHCas and RHHFes increases with raising concentration of the metal salts. These results agree with the low O contents of these samples. Low-molecular weight compounds such as water-soluble acids produced from the hydrolysis of biomass catalyzed by the two Lewis acid metal salts could be dissolved in water and this process could generate pore structures on the hydrochar products. These results also indicate that higher Lewis acidic metal salt concentration could be favorable for pore expansion rather than pore forming to obtain larger pore sizes of the final hydrochars. The relatively high BET surface areas, large pore volumes and big pore diameters of the rice husk hydrochars from metal chloride-assisted HTC suggest their potential application as adsorbents for the removal of organic pollutants.

As could be observed from the thermogravimetric analysis curves of the hydrochars (Additional file 1: Fig. S1), the thermal decomposition process of the rice husk hydrochars can be divided into three stages: the weight loss around 100 °C related to the evaporation of moisture and volatile components, the decomposition of cellulose and hemicellulose below 400 °C, and lignin decomposition (Shi et al. 2018). The presence of different metal chlorides in the aqueous medium reveals different influences on the thermal stability of the hydrochars. The TGA curves of RHHKs are similar to that of RHH. RHHCa0.2 lost the most weight (over 95%) among all the hydrochars, indicating CaCl_2_ at low concentration might decrease the thermal stability of the hydrochars. On the other hand, as compared to RHH, the decomposition of RHHCa0.6, RHHCa1 and RHHFes became lower as the temperature rose, especially below 700 °C, reflecting a better thermal stability of these samples, which could be associated with their higher aromatization.

**Fig. 2:**
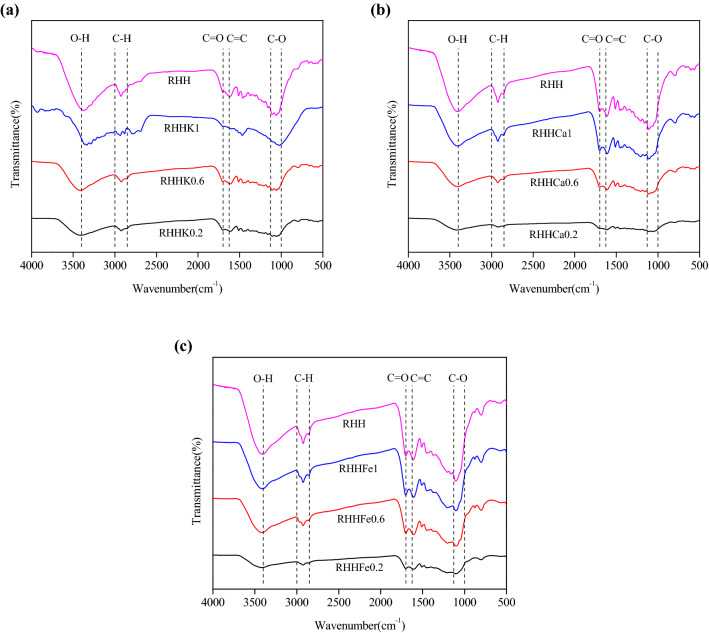
FTIR spectra of rice husk hydrochar sample **a** RHH and RHHKs; **b** RHH and RHHCas; and **c** RHH and RHHFes

According to the FTIR spectra of RHHs (Fig. 2), all the hydrochar samples reveal rich and similar surface functional groups including O–H (around 3400 cm^−1^), C=O (around 1700 cm^−1^), C=C (around 1625 cm^−1^), C–O (between 1130 and 1000 cm^−1^) and C–H (between 3000 and 2850 cm^−1^), and additives in feed water do not show remarkable influences on the types of functional groups on the hydrochars’ surfaces. Surface functional groups especially O-containing functional groups could assist chemical interaction involved in adsorption of organics, and these results further indicate that the metal chloride-assisted HTC chars could be potential adsorbents to remove organic pollutants from wastewater.

**Fig. 3:**
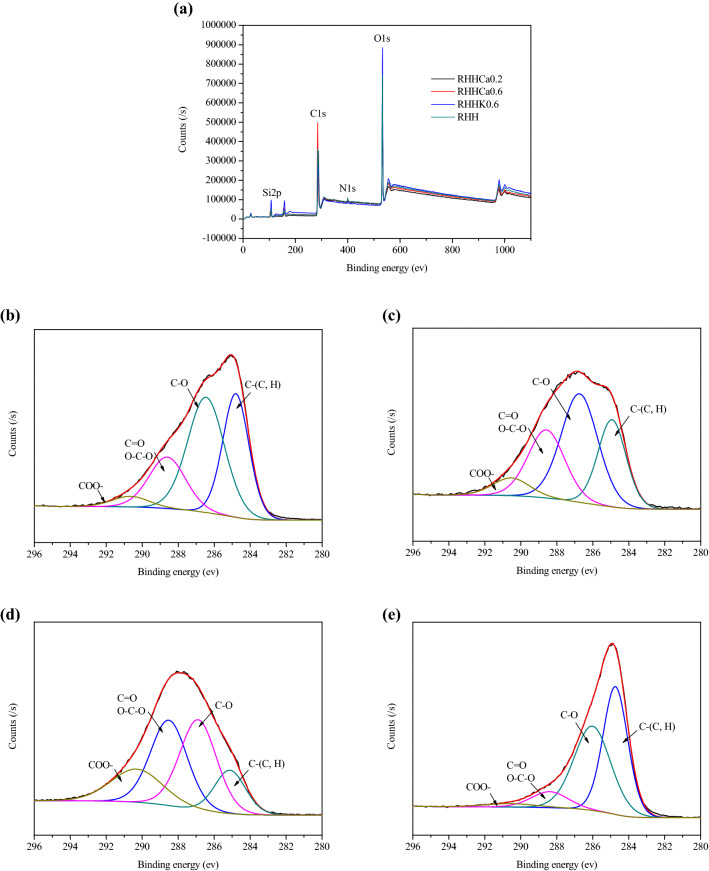
**a** XPS survey; C1s spectra of hydrochar sample; **b** RHH; **c** RHHK0.6; **d** RHHCa0.2 and **e** RHHCa0.6

The surface chemical composition and C1s spectra of the selected hydrochar samples analyzed by XPS are shown in Fig. 3. The survey spectra (Fig. 3a) shows that the hydrochars’ surfaces mainly contains carbon (284.8 eV) and oxygen (531.8 eV), and a small amount of nitrogen and silica (Zhu et al. 2019). As shown in Fig. 3b–e, similar functional groups including C–(C, H) (near 284.5 eV), C–O (near 286 eV), C=O or O–C–O (near 287 eV) and COO– (near 288.5 eV) are observed, indicating the presence of graphite-like carbon and a large amount of O-containing functional groups such as hydroxyl groups, ethers, carboxyl and ester groups on the hydrochars. On the other hand, the hydrochars display different carbon environments, as presented in Table 2, suggesting the dependence of the carbon functionalities on the metal chlorides added. RHHK0.6 and RHHCa0.2 show lower C–(C, H) and C–O contents and higher C=O (O–C–O) and COO– contents than RHH, which could be attributed to the severer hydrothermal decomposition of rice husk structural components in the metal chloride-added HTC environment, while CaCl_2_ represents a more notable effect than KCl. Contrarily, compared to RHH, the intensity of C–(C, H) shows a significant increase in RHHCa0.6, while its C=O (O–C–O) and COO– concentrations decrease significantly, implying a higher hydrophobic surface of this sample.

**Table 2:** Relative atomic concentration of C in the selected hydrochars

Sample	C–(C, H) (%)	C–O (%)	C=O O–C–O (%)	COO– (%)
RHH	33.2	43.5	19.4	4.0
RHHK0.6	25.6	41.6	26.1	6.8
RHHCa0.2	14.2	34.9	33.0	17.9
RHHCa0.6	45.0	44.2	8.4	2.5

**Fig. 4:**
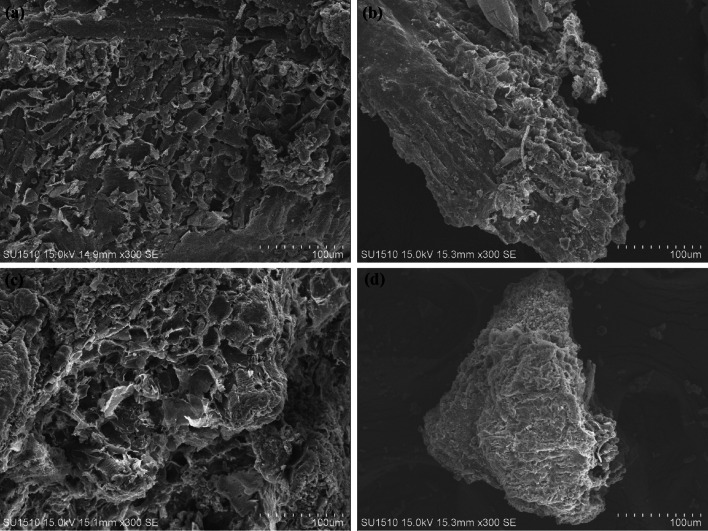
SEM images of **a** rice husk; **b** sample RHH; **c** sample RHHK0.6 and **d** sample RHHCa0.6

SEM images of rice husk feedstock and the selected hydrochar samples (Fig. 4) show that rice husk has natural pore structures on its surface, which could still be maintained after hydrothermal carbonization. On the other side, sample RHHCa0.6 displays a rougher surface than RHH and RHHK0.6, which might serve more sites for the adsorption of organics, and this result agrees with the BET specific surface areas of these samples.

### Adsorption thermodynamics

**Fig. 5:**
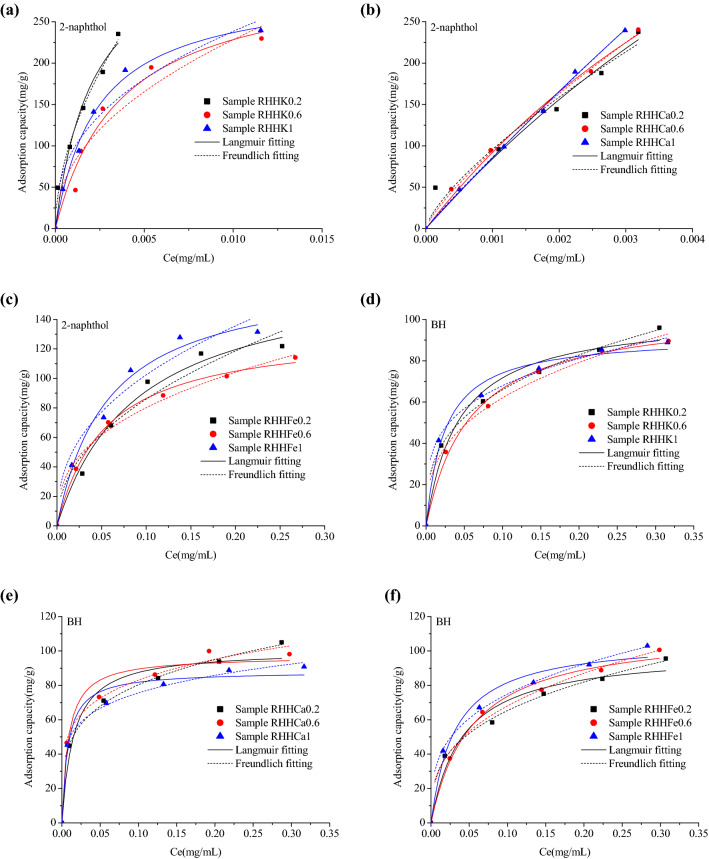

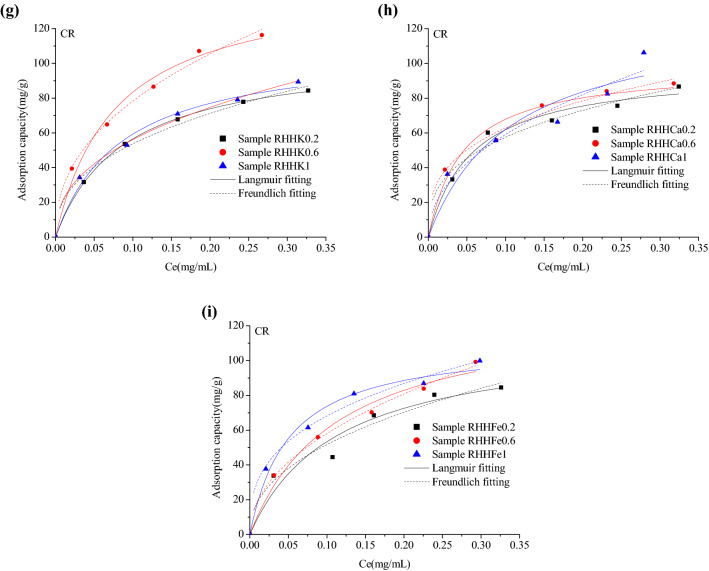
Experimental adsorption isotherm data and fitting curves of **a**–**c** 2-naphthol; d-f BH and **g**–**i** CR on RHHKs, RHHCas and RHHFes at 298 K

Adsorption isotherms of 2-naphthol, BH and CR were tested to depict the adsorption phenomena and elucidate the adsorption mechanism of organics on the rice husk hydrochars. The adsorption isotherm plots and model fitting curves of the three organics on RHHs are displayed in Fig. 5, and the Langmuir and Freundlich parameters as well as the correlation coefficients were calculated and summarized in Additional file 1: Table S2–4. It is observed that RHHs process different adsorption capabilities for the three organics and provide the largest adsorption capacities for 2-naphthol at the same equilibrium concentration. The calculated maximum adsorption capacities from Langmuir model for 2-naphthol are in the range of 170.1–2680 mg/g (Additional file 1: Table S2), some of the values are much higher than those on RHH (61.5 mg/g at the initial concentration of 0.5 mg/mL) and some reported carbonaceous adsorbents derived from biomass such as bamboo hydrochars (with calculated maximum adsorption amount of 322.6 mg/g) (Li et al. 2018), rice straw hydrochars (with maximum adsorption value of 174.9 mg/g) (Li et al. 2019b) and sewage sludge-based activated carbon (with calculated adsorption capacity of 111.9 mg/g) (Gu et al. 2013). 2-Naphthol has a high octanol–water partition coefficient of log *K*_ow_ = 2.7 (Gu et al. 2013), which indicates its higher hydrophobicity. Compared to bamboo hydrochars and rice straw hydrochars, RHHs prepared in this study present higher C contents indicating higher hydrophobicity of their surfaces, which could be favorable for removing aromatic pollutants like 2-naphthol from water through hydrophobic interactions. In addition, hydrogen bonding could be formed between the formaldehyde carbonyl groups on the hydrochar surface and the phenolic hydroxyl groups of 2-naphthol (He et al. 2010), which is also one of the main driving forces for the adsorption of organics. These might be the reasons for the efficient adsorption of 2-naphthol on the rice husk hydrochars. The adsorption amounts of 2-naphthol onto RHHCas are much enhanced than those on RHHKs and RHHFes, additionally, the near-linear adsorption isotherms of 2-naphthol on RHHCas (Fig. 5b) indicate a partition involved adsorption mechanism (Chiou et al. 2015). These results suggest a clear influence of different metal chlorides on the adsorption capabilities of the rice husk hydrochars for 2-naphthol. However, the adsorption capacities of 2-naphthol do not show linear correlations with the elemental compositions or pore properties of RHHs, reflecting a complex relationship between the physico-chemical properties of the hydrochars and their adsorption abilities for organics.

The theoretic adsorption amounts calculated from Langmuir model for BH and CR on RHHs are 88.16–112.7 mg/g and 96.76–147.3 mg/g (Additional file 1: Tables S3, S4), respectively, which are equivalent to the experimental adsorption amounts on RHH (67.5 mg/g for BH and 110.1 mg/g for CR at the initial concentration of 0.5 mg/mL), but lower than the adsorption values for both the adsorbates on rice straw hydrochars prepared through microwave-assisted HTC (Li et al. 2019b). BH is a cationic plant alkaloid, while CR is an anionic azo dye, both of them exist as ions in aqueous solutions, whereas the relatively higher hydrophobicity of RHHs prepared in this work should not benefit the electrostatic attraction between BH, CR and the surfaces of the rice husk hydrochars, which might be responsible for the lower adsorption abilities of RHHs for the two adsorbates. Moreover, all the rice husk hydrochars exhibit similar adsorption capabilities for BH and CR. The oxygen contents of the rice husk hydrochars are approximately in inverse proportion to their specific surface areas (Table 1), while the zeta potential values do not have linear correlations with their elemental compositions or pore properties. Oxygen-containing group could benefit chemical and semi-chemical adsorption of organics, zeta potential could influence electrostatic attraction of the hydrochars for organic ions, while high specific surface area indicates rich adsorption sites, and the similar adsorption capacities of BH and CR could be attributed to the combined effect of surface chemistry and pore texture of the chars.

The adsorption isotherm data of all the three organics can be well fitted by both Langmuir model (*R*^2^ > 0.97 for 2-naphthol, *R*^2^ > 0.96 for BH and *R*^2^ > 0.92 for CR) and Freundlich model (*R*^2^ > 0.93 for 2-naphthol, *R*^2^ > 0.99 for BH and *R*^2^ > 0.96 for CR). These results indicate that the adsorption of the organics on RHHs can be considered as monolayer adsorption on heterogeneous surfaces. The 1/n value from Freundlich model is less than 1 for all the three organics on the rice husk hydrochars, implying a favorable adsorption process.

**Fig. 6:**
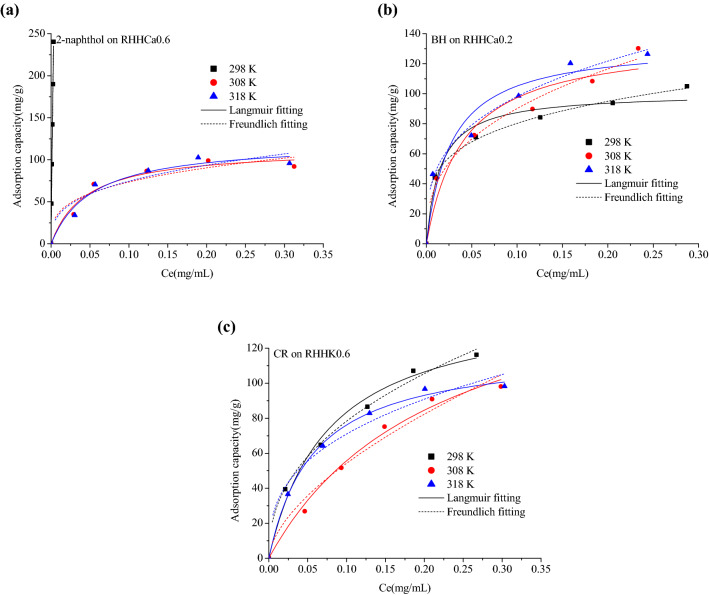
Experimental adsorption isotherm data and fitting curves of **a** 2-naphthol on RHHCa0.6; **b** BH on RHHCa0.2; **c** CR on RHHK0.6 at 298, 308 and 318 K

RHHCa0.6, RHHCa0.2 and RHHK0.6 have higher yields and show larger adsorption capacities for the three organics than all the other rice husk hydrochars, thus were employed as the adsorbents for the following tests. Figure 6 gives the adsorption isotherm data and model fitting curves of 2-naphthol on RHHCa0.6, BH on RHHCa0.2 and CR on RHHK0.6 at 298, 308 and 318 K, and the model fitting parameters are listed in Additional file 1: Table S5. It is obvious that the adsorption capacity of 2-naphthol onto RHHCa0.6 sharply decreases with increasing temperature from 298 to 308 K, while the K_L_ also decreases, implying the weaker adsorption driving force at a higher temperature, and these results suggest that physisorption through weak intermolecular interactions such as hydrophobic interactions is the dominant adsorption mechanism in this case and the adsorption process is exothermic. Unlike the adsorption of 2-naphthol, the adsorption amounts of BH onto RHHCa0.2 and CR on RHHK0.6 do not present linear correlations with the feed temperatures. RHHCa0.2 provides similar adsorption capabilities for BH at different temperatures when the equilibrium concentration is lower than 0.05 mg/mL, then the adsorption capacity increases with rising temperature at medium concentrations (from 0.05 to 0.25 mg/mL), and when the concentration is higher than 0.25 mg/mL, the adsorption amount increases from 298 to 308 K, then decreases from 308 to 318 K. On the other hand, the adsorption amount of CR on RHHK0.6 decreases with increasing feed temperature from 298 to 308 K, then increases from 308 to 318 K. These results reveal a chemisorption-involved adsorption mechanism which might be attributed to the chemical or semi-chemical interactions between the oxygen-containing functional groups on the hydrochars’ surfaces and the two organics.

The thermodynamic parameters including adsorption enthalpy ∆H (kJ/mo1), adsorption free energy change ∆G (kJ/mo1) and adsorption entropy ∆S (J/(mo1 K)) calculated based on the Van’t Hoff equation (Gupta et al. 2010) for the three organics on the selected hydrochars are listed in Table 3. The ∆G values are negative and between − 20 kJ/mol and 0 kJ/mol, indicating a thermodynamically favorable adsorption process with physical interactions as the main adsorption driving force for all the organics on the rice husk hydrochars (Baseri et al. 2012). The negative ∆H values imply an exothermic adsorption process for all the adsorbates on the selected hydrochars. The positive ∆S values for BH and CR suggest the whole system is disordered after adsorption, which reflects the irreversibility of the adsorption process for both the adsorbates. On the other hand, the negative ∆S for the adsorption of 2-naphthol denotes the decrease in randomness of the adsorption system, together with the negative ∆H, the adsorption of 2-naphthol on RHHCa0.6 should be an enthalpy-driven adsorption process.

**Table 3:** Adsorption thermodynamic parameters of the three organics on the selected hydrochars

Sample	Δ*G*(kJ/mol)			Δ*H* (kJ/mol)	Δ*S* (KJ/mol K)
	298 K	308 K	318 K		
2-Naphthol on RHHCa0.6	− 6.094	− 5.092	− 5.238	− 18.66	− 0.043
BH on RHHCa0.2	− 5.143	− 5.300	− 5.222	− 4.002	0.004
CR on RHHK0.6	− 4.959	− 4.801	− 5.244	− 0.612	0.014

### Adsorption kinetics

**Fig. 7:**
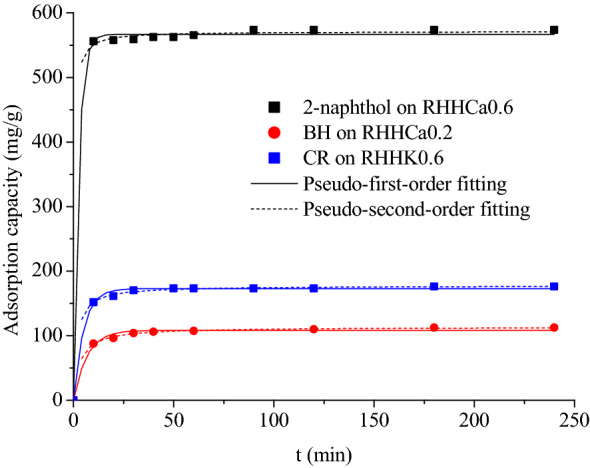
Adsorption kinetics of **a** 2-naphthol on RHHCa0.6; **b** BH on RHHCa0.2 and **c** CR on RHHK0.6 at 298 K

Figure 7 displays the experimental adsorption kinetics and model fitting curves of the three organics onto the selected rice husk hydrochars from aqueous solutions at 298 K, and the fitting parameters were calculated and listed in Additional file 1: Table S6. The time required for the adsorption of 2-naphthol onto RHHCa0.6, BH onto RHHCa0.2 and CR onto RHHK0.6 to reach equilibrium is about 10, 40 and 30 min, respectively, reflecting a fast adsorption rate of all the three adsorbates on the hydrochars which should be due to the large pore sizes of these hydrochar samples (Table 1).

It is noted from the fitting results listed in Additional file 1: Table S6 that both the pseudo-first-order equation (*R*^2^ > 0.990) and pseudo-second-order equation (*R*^2^ > 0.999) can characterize the adsorption kinetics of the organics very well. The calculated *v*_0_ value from the pseudo-second-order equation is 1544, 36.78 and 104.6 mg/(g min) for 2-naphthol, BH and CR, respectively, which is in accordance with the experimental observations of the required time to establish adsorption equilibrium. Both the equations assume that the adsorption process is controlled by the mass action (adsorption reaction) at the liquid/solid interface (Simonin 2016). The rice husk hydrochars exhibit much lower BET specific surface areas (Table 1) than porous carbonaceous materials such as commercial activated carbon, undeveloped inner surface and large pore size of the hydrochars could reduce the resistance to intra-particle diffusion, thus mass action could be regarded as the rate-controlling step of the adsorption, and this may explain the fast kinetics of the three organics on the hydrochars and the good fitting results from the two adsorption kinetic equations.

## Conclusion

The present study demonstrates that it is possible to produce rice husk hydrochars as effective adsorbents for removing organics from aqueous solution through metal chloride-assisted hydrothermal carbonization. The addition of metal chlorides in feed solution clearly affects the yield, ash content, elemental composition (C content: 45.01–58.71%), zeta potential, thermal stability, surface O-containing functional groups and pore properties (surface area: 13.23–45.97 m^2^/g) of the chars and their adsorption efficacies for organics. The calculated maximum adsorption capacities of 2-naphthol are ranging from 170.1 to 2680 mg/g, much higher than those of berberine hydrochloride and Congo red on the hydrochars. The adsorption of the organics on the selected hydrochars is a spontaneous physisorption-dominated process, and the adsorption is fast with equilibrium time less than 30 min and controlled by the mass action step kinetically. These results provide a reference to the treatment of agricultural residue wastes, and suggest rice husk hydrochars as a promising material to remove pollutant organics from water.

### Supplementary Information

**Additional file 1.** Supplementary material.

## Data Availability

All data generated or analyzed during this study are included in this published article (and its Additional files).

## Abbreviations

HTC: Hydrothermal carbonization
RH: Rice husk
BH: Berberine hydrochloride
CR: Congo red
XRD: X-ray diffraction
FTIR: Fourier transform infrared
BET: Brunauer–Emmett–Teller
TGA: Thermogravimetric analysis
XPS: X-ray photoelectron spectroscopy
SEM: Scanning electron microscope
HMF: 5-Hydroxymethylfurfural
