# Supplementary material for: Rice husk hydrochars from metal chloride-assisted hydrothermal carbonization as biosorbents of organics from aqueous solution
Source: Bioresour Bioprocess. 2021 Oct 9;8(1):99. doi: 10.1186/s40643-021-00451-w (PMC10991232; doi:10.1186/s40643-021-00451-w)
Supplement: Supplementary file 1 — Additional file 1. Supplementary material. [file 40643_2021_451_MOESM1_ESM.doc]

**Rice Husk Hydrochars from Metal Chloride Assisted Hydrothermal Carbonization as Biosorbents of Organics from Aqueous Solution**

Yin Li ^a^, *, Fana Hagos Mulugeta ^a^, Rongrong Chen ^a^, Hanxin Qian^a^, Chengxing Mo^a^, Jing Di ^a^, Xikun Gai ^a^, *, Ruiqin Yang ^a^, Genxing Pan ^b^, Shengdao Shan ^c^

*^a^* *Zhejiang Provincial Key Lab for Chemical and Biological Processing Technology of Farm Product, School of Biological and Chemical Engineering, Zhejiang University of Science and Technology, Hangzhou 310023, Zhejiang, China*

*^b^Institute of Resource, Ecosystem and Environment of Agriculture, Nanjing Agricultural University, 1 Weigang, Nanjing 210095, China*

*^c^ Key Laboratory of Recycling and Eco-treatment of Waste Biomass of Zhejiang Province, Zhejiang University of Science and Technology, Hangzhou 310023, Zhejiang, China*

**Supplementary material**

* Corresponding author.

* Yin Li, Tel: 86-571-85070380. E-mail address: cherryli1986@126.com (Y. Li)

* Xikun Gai, Tel: 86-571-85070196. E-mail address: gaixikun@163.com (X. Gai)

Table S1. Formula, chemical structure and molecular weight of 2-naphthol, berberine hydrochloride and Congo red

| Compound | Formula | Molecular structure | Molecular weight |
| --- | --- | --- | --- |
| 2-naphthol | C_10_H_7_OH |  | 144.17 |
| Berberine hydrochloride | C_20_H_18_ClNO_4_ |  | 371.8 |
| Congo red | C_32_H_22_N_6_Na_2_O_6_S_2_ |  | 696.66 |

Table S2. Langmuir and Freundlich fitting parameters of experimental adsorption isotherm data for 2-naphthol at 298 K

| Hydrochar sample | Langmuir fitting | | | Freundlich fitting | | |
| --- | --- | --- | --- | --- | --- | --- |
|  | *Q*_m_  (mg/g) | *K*_L_  (mL/mg) | R^2^ | *K*_F_ (mg/g)(mL/mg)^1/n^ | 1/n | R^2^ |
| RHHK0.2 | 361.6 | 461.9 | 0.975 | 4880 | 0.543 | 0.995 |
| RHHK0.6 | 311.7 | 274.1 | 0.972 | 1945 | 0.465 | 0.934 |
| RHHK1 | 293.7 | 417.6 | 0.994 | 1495 | 0.399 | 0.960 |
| RHHCa0.2 | 1051 | 87.06 | 0.960 | 1.488×10^4^ | 0.730 | 0.971 |
| RHHCa0.6 | 808.1 | 128.5 | 0.994 | 2.272×10^4^ | 0.794 | 0.998 |
| RHHCa1 | 2680 | 32.98 | 0.998 | 5.632×10^4^ | 0.938 | 0.998 |
| RHHFe0.2 | 173.3 | 11.12 | 0.988 | 250.2 | 0.464 | 0.959 |
| RHHFe0.6 | 133.3 | 18.43 | 0.997 | 191.4 | 0.378 | 0.992 |
| RHHFe1 | 171.0 | 17.56 | 0.988 | 260.5 | 0.406 | 0.967 |

Table S3. Langmuir and Freundlich fitting parameters of experimental adsorption isotherm data for berberine hydrochloride at 298 K

| Hydrochar sample | Langmuir fitting | | | Freundlich fitting | | |
| --- | --- | --- | --- | --- | --- | --- |
|  | *Q*_m_  (mg/g) | *K*_L_  (mL/mg) | R^2^ | *K*_F_ (mg/g)(mL/mg)^1/n^ | 1/n | R^2^ |
| RHHK0.2 | 102.4 | 23.40 | 0.980 | 140.5 | 0.328 | 0.999 |
| RHHK0.6 | 105.0 | 17.30 | 0.996 | 139.3 | 0.351 | 0.994 |
| RHHK0.1 | 92.22 | 40.88 | 0.988 | 122.3 | 0.256 | 0.998 |
| RHHCa0.2 | 100.7 | 66.08 | 0.971 | 140.1 | 0.242 | 0.999 |
| RHHCa0.6 | 96.95 | 119.8 | 0.974 | 130.5 | 0.196 | 0.992 |
| RHHCa1 | 88.16 | 128.4 | 0.981 | 115.1 | 0.182 | 0.998 |
| RHHFe0.2 | 100.3 | 24.41 | 0.969 | 138.7 | 0.327 | 0.998 |
| RHHFe0.6 | 112.7 | 18.83 | 0.991 | 154.2 | 0.355 | 0.993 |
| RHHFe1 | 106.9 | 32.25 | 0.982 | 150.0 | 0.301 | 0.999 |

Table S4. Langmuir and Freundlich fitting parameters of experimental adsorption isotherm data for Congo red at 298 K

| Hydrochar sample | Langmuir fitting | | | Freundlich fitting | | |
| --- | --- | --- | --- | --- | --- | --- |
|  | *Q*_m_  (mg/g) | *K*_L_  (mL/mg) | R^2^ | *K*_F_ (mg/g)(mL/mg)^1/n^ | 1/n | R^2^ |
| RHHK0.2 | 106.4 | 11.33 | 0.999 | 137.1 | 0.407 | 0.992 |
| RHHK0.6 | 147.3 | 13.05 | 0.989 | 210.8 | 0.430 | 0.996 |
| RHHK1 | 109.9 | 11.70 | 0.990 | 145.0 | 0.412 | 0.998 |
| RHHCa0.2 | 96.76 | 17.72 | 0.987 | 126.7 | 0.343 | 0.982 |
| RHHCa0.6 | 98.06 | 22.46 | 0.974 | 131.4 | 0.321 | 0.990 |
| RHHCa1 | 128.6 | 9.304 | 0.926 | 175.7 | 0.473 | 0.963 |
| RHHFe0.2 | 113.4 | 8.901 | 0.947 | 143.1 | 0.442 | 0.974 |
| RHHFe0.6 | 130.2 | 8.770 | 0.984 | 174.7 | 0.479 | 0.998 |
| RHHFe1 | 110.9 | 19.85 | 0.986 | 151.8 | 0.348 | 0.994 |

Table S5. Langmuir and Freundlich fitting parameters of experimental adsorption isotherm data for organics on selected hydrochars at different temperatures

| Sample | T(K) | Langmuir fitting | | | Freundlich fitting | | |
| --- | --- | --- | --- | --- | --- | --- | --- |
|  |  | *Q*_m_  (mg/g) | *K*_L_  (mL/mg) | R^2^ | *K*_F_ (mg/g)(mL/mg)^1/n^ | 1/n | R^2^ |
| 2-naphthol on RHHCa0.6 | 298 | 808.1 | 128.5 | 0.994 | 2.272×10^4^ | 0.794 | 0.998 |
|  | 308 | 113.9 | 22.53 | 0.965 | 145.8 | 0.297 | 0.919 |
|  | 318 | 121.9 | 18.92 | 0.964 | 158.9 | 0.329 | 0.920 |
| BH on RHHCa0.2 | 298 | 100.7 | 66.08 | 0.971 | 140.1 | 0.242 | 0.999 |
|  | 308 | 136.6 | 24.82 | 0.950 | 211.0 | 0.369 | 0.990 |
|  | 318 | 133.6 | 36.85 | 0.941 | 200.8 | 0.311 | 0.990 |
| CR on RHHK0.6 | 298 | 147.3 | 13.05 | 0.989 | 210.8 | 0.430 | 0.996 |
|  | 308 | 175.8 | 4.639 | 0.991 | 216.6 | 0.601 | 0.977 |
|  | 318 | 119.7 | 17.50 | 0.997 | 161.1 | 0.356 | 0.979 |

Table S6. Fitting results from Pseudo-first-order and Pseudo-second-order models for adsorption kinetics of the three organics on selected hydrochars at 298 K.

| Sample | Pseudo-first-order | | | Pseudo-second-order | | | |
| --- | --- | --- | --- | --- | --- | --- | --- |
|  | *q*_e_ (mg/g) | *k*_1_ (1/min) | R^2^ | *q*_e_ (mg/g) | *k*_2_ | *v*_0_ (mg/ (g.min)) | R^2^ |
| 2-naphthol on RHHCa0.6 | 566.8 | 0.3909 | 0.999 | 571.4 | 4.730×10^-3^ | 1544 | 0.999 |
| BH on RHHCa0.2 | 108.4 | 0.1492 | 0.990 | 113.6 | 2.850×10^-3^ | 36.78 | 0.999 |
| CR on RHHK0.6 | 172.8 | 0.2004 | 0.996 | 177.5 | 3.320×10^-3^ | 104.6 | 0.999 |

(a)

(b)

(c)

Fig. S1 TG and DTG curves of rice husk hydrochar sample (a) RHH and RHHKs; (b) RHH and RHHCas; and (c) RHH and RHHFes
